# When infection prevention enters the temple: Intergenerational social distancing and COVID-19

**DOI:** 10.1017/ice.2020.100

**Published:** 2020-04-01

**Authors:** David M. Hartley, Heather Schacht Reisinger, Eli N. Perencevich

**Affiliations:** 1 Cincinnati Children’s Hospital, James M. Anderson Center for Health Systems Excellence, Cincinnati, Ohio; 2 Department of Pediatrics, University of Cincinnati, College of Medicine, Cincinnati, Ohio; 3 Center for Access & Delivery Research and Evaluation, Iowa City Veterans’ Affairs Health Care System, Iowa City, Iowa; 4 Department of Internal Medicine, Carver College of Medicine, University of Iowa, Iowa City, Iowa

*To the Editor*—The recent emergence of SARS-CoV-2 and the pandemic of associated COVID-19 disease poses significant though incompletely determined threats to human health globally. Although uncertainties predominate the epidemiology of this new virus,^1^ several observations are relevant for policy making at this stage of the pandemic:

- Asymptomatic transmission. Increasing evidence suggests that the time from infection to infectiousness (the latency period) is less than the time to symptoms (the incubation period). Thus, people may inadvertently infect others before realizing they are infected because they are not yet experiencing symptoms. Asymptotic cases may be particularly common in children and young adults.
- Disease severity and risk of death. The crude case fatality ratio is estimated to be significantly higher than that for seasonal influenza,^3^ and it is dramatically higher in the aged and those with underlying comorbid conditions (especially cardiovascular disease, pulmonary disease, and diabetes) regardless of age.^3^ Persons in at-risk strata form a significant fraction of the world population.
- Lack of options for control. No vaccine or effective antiviral drug is likely to be widely available for months or longer.^4^

Combined with lack of widespread diagnostic testing, these factors have produced one crucial implication for public health: Without intervention, people in the high-risk strata will be exposed by those around them who do not realize they are infectious. This situation is particularly acute with intergenerational mixing among the asymptomatic, in which infectious youths might intermingle with the high-risk elderly.

Given the lack of a vaccine and drugs for treatment, how do we minimize the community risk of becoming infected? Nonpharmaceutical interventions (eg, broad-scale social distancing, including school closures, working from home, and limiting large-sized gatherings) are needed to minimize transmission. Analyses are demonstrating the theoretical and historical impacts of such measures in scenarios similar to what we face now.^5^

Importantly, large intergenerational gatherings, including religious services, have amplified the spread of SARS-CoV-2 in South Korea, Malaysia, and other countries.^6–9^ Traditions such as handshaking, embracing, touching the Torah, use of prayer mats and passing offering plates, for example, could place persons at risk for acquiring SARS-CoV-2 in close proximity to those who may be asymptomatically or inapparently infectious. Some religious communities have recently offered guidance to congregations aimed at minimizing risk of transmission, including suspending in-person services for weeks.

Such policies must be implemented immediately. Waiting until community transmission is detected—according to any definition—is too late,^10^ even if surveillance systems capable of detecting transmission with any degree of sensitivity or timeliness existed, which they do not. The difference in latency versus incubation period in this novel pathogen obviates the appropriateness of such a policy. The time for social distancing is now.
